# Clinical Characteristics, Laboratory Findings, Management, and Outcome of Severe Coronavirus Disease 2019 in Children at a Tertiary Care Center in Riyadh, Saudi Arabia: A Retrospective Study

**DOI:** 10.3389/fped.2022.865441

**Published:** 2022-05-03

**Authors:** AbdulAziz AlMayouf, Dayel AlShahrani, Salwan AlGhain, Sarah AlFaraj, Yara Bashawri, Tariq AlFawaz, Egab AlDosari, Laila Al-Awdah, Mohammed AlShehri, Yara AlGoraini

**Affiliations:** ^1^King Fahd Medical City, Riyadh, Saudi Arabia; ^2^King Faisal Specialist Hospital & Research Centre, Riyadh, Saudi Arabia; ^3^Prince Mohammed Bin Abdulaziz Hospital, Riyadh, Saudi Arabia

**Keywords:** COVID-19, characteristics, management, outcome, children, coronavirus disease

## Abstract

**Background:**

Numerous studies worldwide have reported COVID-19 in children; however, the clinical symptoms and consequences of COVID-19 in children have only been reported in a few studies in Saudi and gulf region. Therefore, we aimed to investigate the clinical features and outcomes of COVID-19 infection in children and the therapeutic interventions used.

**Methods:**

This retrospective cohort study included 96 patients with confirmed severe acute respiratory syndrome coronavirus 2 infection aged ≤14 years who were admitted to a tertiary governmental care hospital in Riyadh, Saudi Arabia between March 2020 and November 2020. Data on children with COVID-19, including demographics, comorbidities, symptoms, imaging and laboratory results, therapies, and clinical outcomes, were analyzed.

**Results:**

Of 96 children admitted with a confirmed diagnosis of COVID-19, 63.8% were aged ≤ 3 years, 52.1% were male, 56.2% had an unknown source of infection, and 51% had no comorbidities. Most cases had severe infection (71.88%) as they required oxygen, 10.42% of whom were critical. The most common symptoms were respiratory-related (98%), and the common physical sign was fever (49%). High D-dimer (90.7%) and C-reactive protein (72.09%) levels were found in most cases. Oxygen (71.88%) was the most commonly used treatment. Most patients were discharged home and fully recovered (97.92%). We reported two deaths (2.08%).

**Conclusions:**

Our findings showed that the majority of the admitted children with COVID-19 were ≤3 years of age (52.1%) and infected with an unknown source (56.2%). Moreover, the majority of the cases had severe COVID-19 infection as they required oxygen (71.88%), although they had favorable outcomes. However, some cases were critical and resulted in death. Future studies will be crucial to better understand the disease spectrum and potential therapeutic options for COVID-19 in children.

COVID-19, coronavirus disease 2019; SA, Saudi Arabia; ICU, intensive care unit; MIS-C, multisystem inflammatory syndrome in childhood; KD, Kawasaki disease; SARS-CoV-2, severe acute respiratory syndrome coronavirus 2; SMOH, Saudi Ministry of Health; PICU, pediatric intensive care unit; ECG, electrocardiogram; CRP, C-reactive protein; ALT, alanine aminotransferase; AST, aspartate aminotransferase.

## Introduction

Numerous studies worldwide have reported COVID-19 with different variants such as omicron, delta, and SARS CoV-2 infection in children. China has published one of the first comprehensive studies, which included 728 patients with SARS CoV-2 infection variant (1). Almost 12% of the patients had no symptoms, 43% had mild symptoms, 40% had moderate symptoms, and 2.9% had severe symptoms or were in critical condition. Children <1 year of age accounted for a larger proportion of severe or critical cases (1). Similarly, one systemic review reported 7,780 pediatric patients with proven SARS CoV-2 infection. Fever and cough (59.1 and 55.9%, respectively) were the most common symptoms of COVID-19 in children, and only 19.3% of patients were asymptomatic (2). Children with comorbidities were the largest subset of COVID-19 cases. Most patients (88.9%) recovered, a minority of patients were admitted to the intensive care unit (ICU) (3.3%), and seven deaths were reported (0.09%) (2). Furthermore, according to a study on the clinical characteristics of COVID-19 in children, fever, cough, and gastrointestinal involvement were far more common in children than in adults (3, 4). The condition is mild for most children, according to these COVID-19 studies in children, with a vast number of children remaining asymptomatic. However, MIS-C represents a subpopulation of children who are more prone to acquiring major disorders related to COVID-19 (2, 5). In a recent study of MIS-C in children and adolescents, 186 cases were discovered across the United States. The individuals' average age was 8.3 years, and 75% of them had previously been healthy. Aneurysms in the coronary arteries were found in 8% of patients, and 40% of them had symptoms similar to KD (5). Additionally, a systematic review and meta-analysis study showed that a high proportion of children with MIS-C were admitted to the ICU (47.1%), and 4.8% of hospitalized children with MIS-C died (4). Moreover, a study in SA showed that five patients diagnosed with MIS-C-associated SARS CoV-2 infection with severe clinical presentation were admitted to the ICU, where they received intravenous immunoglobulin and inotropic support (6).

There are few studies in SA and the Gulf region, on the clinical characteristics and consequences of COVID-19 in children (7–14). However, COVID-19 predisposing factors affecting Saudi adults such as diabetes, obesity and vitamin D deficiency has been documented (8–10). In a study of SARS CoV-2 infection in SA, 4.8% of cases were in children aged ≤14 years (11). The clinical features and outcomes of these pediatric patients, on the other hand, were not documented (11). However, one of the initial studies from SA detailed the clinical aspects of COVID-19 in children, revealing that 54% of the cases were male, with an average age of 8.4 years, and 75% had normal white blood cell counts and eosinopenia (15). In another similar study in SA, children infected with SARS CoV-2 had a median age of almost 7 years, and the most prevalent symptom was fever (32.5%), followed by respiratory symptoms (21%), and gastrointestinal symptoms (10.3%) (6). Seven patients with COVID-19-related symptoms were admitted to the ICU, five of whom had multisystem inflammatory syndrome in childhood (MIS-C), one had Kawasaki disease (KD), and one had pneumonia (6).

In the other hand, the latest infectious variant is Omicron, which was found to be more transmissible and less pathogenic than previously circulating variants (16). On December 1, 2021, the Omicron variant was first clinically diagnosed in the United States, and it quickly spread (16). Despite the fact that Omicron had the most COVID-19 cases and hospitalizations during the pandemic, illness severity measures such as duration of stay, ICU admission, and mortality were lower than during past pandemic peaks (16). This highlights the need of national disaster preparation when critical care demands occur before the system becomes overloaded. In addition, staying up to date on COVID-19 immunizations and implementing other suggested COVID-19 preventative practices are crucial to preventing infections, serious illness, or death from COVID-19 among unvaccinated people (16). Health care workers (HCWs) in SA showed a high conviction in SARS-CoV-2 Omicron Variant infection prevention measures (17). Moreover, increase stress among HCWs in SA immediately after a new infectious threat emerges (18). The Omicron variant is causing a new wave of the COVID-19 outbreak over the world. The origin, pathogenicity, virulence, and immunogenic escape of this variation are all yet unknown (19).

There are limited data available on the characteristics of severe SARS CoV-2 infection of COVID-19 in children in SA and the Gulf region despite there are many descriptive studies in children have been performed internationally. Therefore, this study aimed to investigate the clinical features and outcomes of severe COVID-19 presentation in children and the therapeutic interventions used.

## Materials and Methods

### Study Design and Setting

This retrospective cohort study was conducted at a tertiary governmental care hospital in Riyadh, SA. This colossal medical facility comprises four hospitals and four medical centers. The hospital has >200 beds covered by general pediatric, subspecialty, and critical care teams. The necessity for informed consent was waived because this was a retrospective study that used data gathered in routine clinical practice between March 2020 and November 2020. The study was approved by the local Institutional Review Board on January 2020.

### Patients and Testing

We retrospectively screened children aged ≤14 years with confirmed severe SARS CoV-2 infection who required oxygen as per Saudi Ministry of Health (SMOH) with signs and symptoms of respiratory tract infection, fever, headache, body ache, loss of taste or smell, chest pain, skin rashes, and gastrointestinal symptoms who were admitted to the tertiary governmental care hospital in Riyadh, SA between March 2020 and November 2020. The rationale behind choosing children aged ≤14 years as an inclusion criterion is the accepted age for treating pediatric patients in SA. Patients with insufficient data, those who went to the emergency for only a nasopharyngeal swab test, and those who had previously been admitted for the same reason were also excluded. Patients who require oxygen were constituted as severe COVID-19 infection.

To screen for an acute respiratory illness among the suspected cases and determine who required naso/oropharyngeal swab testing, we used the Saudi Center for Disease Prevention and Control “Weqaya” (12) and the Saudi Ministry of Health (SMOH) standard visual triage checklist (13). The checklist asses the exposure risk by multiple questions if the patient has history of travel abroad in the past 14 days, a contact with a confirmed case of SARS CoV-2 infection in the last 14 days prior to symptom onset, an exposure to camel or camel's products in the last 14 days prior to symptom onset, or working in a healthcare facility. Also, the checklist asses the clinical signs and symptoms as fever or history of fever, cough, shortness of breath, headache or sore throat, nausea or vomiting, diarrhea, and if he has any chronic disease. Any patient scored ≥ 4 will be assessed by physician, isolated in a respiratory room, and wear a mask.

To confirm that all study patients were positive for SARS CoV-2 infection, we performed a real-time reverse transcription-polymerase chain reaction test for the qualitative detection of nucleic acids from severe acute respiratory syndrome coronavirus 2 (SARS-CoV-2) from upper respiratory specimens, such as naso/oropharyngeal tissues. All patients provided nasopharyngeal swabs in a child-friendly setting. The patient was considered COVID-19 positive if the initial swab result was positive, or if it was negative at first but positive on a second test. The swab test was repeated soon after the initial results were negative if there was a high clinical suspicion of COVID-19 or a risk of false-negative results due to technological mistakes during sample processing. Some patients had multiple positive swab samples during their stay, as required by the responsible team in accordance with the center's local procedure and revisions in the SMOH's recommendations, which was altered over time based on the most recently published data.

### Data Collection

Data was gathered from patient's electronic health records. Patient demographics (age, sex, and nationality), length of hospital stay, duration of symptoms, potential cause of infection, need for pediatric intensive care unit (PICU) admission, length of stay in the PICU, need for intubation, presenting symptoms and physical signs, and associated comorbidities were all collected. We also considered the possibility of multiorgan failure, acute respiratory disease syndrome, pneumonia, or central nervous system complications caused by COVID-19. The degree of respiratory distress, chest radiography results, and the need for oxygen therapy and/or mechanical ventilation were also considered.

Detailed demographic and clinical data, such as presenting signs and symptoms, length of PICU stay, need for mechanical ventilation, and electrocardiogram (ECG) abnormalities, were collected for any patient who required intensive care admission. Laboratory findings were reported, and the associated hematological abnormalities were assessed, including significant anemia, leukopenia, leukocytosis, neutropenia or neutrophilia, and thrombocytopenia. Other reported laboratory abnormalities included high liver function test results and lactic acid dehydrogenase and D-dimer levels. High acute-phase reactants [C-reactive protein (CRP) and ferritin] and evidence of any related infection (in the blood, urine, and tracheal aspirate) were highlighted. In addition, we recorded any viral respiratory infection that may be present.

Moreover, during the study period, we encountered some cases that fulfilled the MIS-C or pediatric multisystem inflammatory syndrome criteria as set by the Center of Disease Control and Prevention (14), Royal College of Pediatrics and Child Health (20), and the World Health Organization (21); thus, these were included in the study. We recorded their demographic data, presenting symptoms and signs, and severity of respiratory disease using their laboratory findings and outcomes.

In the pediatric age group, we referred to the most used drugs as active COVID-19 therapy. Patients who had completed their hospitalization by the conclusion of the study period, including those who were discharged home or died, had their clinical outcomes recorded. Clinical outcome indicators included the full recovery rate, death rate, length of hospital stay, PICU admission, and the necessity for invasive therapy such as mechanical ventilation.

### Statistical Analysis

It is a descriptive analysis so no relation or association were tested. Continuous variables are presented as mean ± standard deviation, and categorical variables are presented as frequencies and percentages. Statistical analysis was conducted using SPSS version 22 (IBM Corp., Armonk, NY, USA).

### Sample Size

Considering the novelty of the diseases at the time no parameters were used in sample size calculation and opted instead to include all patients who meet the inclusion criteria.

## Results

A 96 children admitted with a confirmed diagnosis of COVID-19 infection, of whom 63.8% were aged ≤ 3 years, 52.1% were male, 96.9% were Saudi, 56.2% had an unknown source of infection to SARS CoV-2, 51% did not have any comorbidities, 74.5% were hospitalized ≤ 10 days due to other comorbidities, and 70.83% had symptoms lasting for ≤3 days (Table 1). Moreover, as per the SMOH protocol, the majority of patients had severe COVID-19 (71.88%) as they required oxygen, 10.42% of whom were critical, and a minority of the cases had moderate COVID-19 (28.12%). Furthermore, most of the children did not develop any complications (80.2%), and oxygen (71.88%) was the most commonly used treatment, followed by antibacterial antibiotics (48.96%). The most common complications associated with COVID-19 were secondary pneumonia (10.4%), followed by multiorgan failure (4.2%), and central nervous system complications (3.1%). Most patients were discharged home (97.92%) and fully recovered (97.92%). Demographic data and disease characteristics are presented in Table 1.

**Table 1:** Patients demographics and disease characteristics associated with the outcome of children admitted with confirmed COVID-19 infection (*n* = 96).

**Variables**		**Total population *n* = 96 (%)**	**Outcome**	
			**Full recovery (%)**	**Death (%)**
			***n* = 94 (97.92%)**	***n* = 2 (2.08%)**
**Age**	≤3	60 (63.8%)	60 (63.8%)	0(0.0%)
	>3	36 (36.20%)	34 (36.2%)	2 (100.0%)
**Sex**	Male	50 (52.1%)	48 (51.1%)	2 (100.0%)
	Female	46 (47.9%)	46 (48.9%)	0(0.0%)
**Nationality**	Saudi	93 (96.9%)	91 (96.8%)	2 (100.0%)
	Non- Saudi	3(3.1%)	3(3.2%)	0(0.0%)
**Length of hospital Stay**	≤10	70 (74.5%)	70 (74.5%)	0(0.0%)
	>10	26 (25.5%)	24 (25.5%)	2 (100.0%)
**Duration of symptoms**	≤3	68 (70.83%)	67 (71.3%)	1 (50.0%)
	>3	28 (29.17%)	27 (28.7%)	1 (50.0%)
**Possible source of infection**	Contact COVID-19 Case	40 (41.67%)	39 (41.5%)	1 (50.0%)
	Travel to endemic area	2 (2.08%)	2(2.1%)	0(0.0%)
	Unknown	54 (56.25%)	53 (56.4%)	1 (50.0%)
**Comorbidities**	Yes	47 (49.0%)	45 (47.9%)	2 (100.0%)
	No	49 (51.0%)	49 (52.1%)	0(0.0%)
^*^Complications	Secondary Pneumonia	10 (10.4%)	9 (90.0%)	1 (10.0%)
	ARDS	2(2.1%)	1(50%)	1(50.0%)
	Multiorgan failure	4(4.2%)	4 (100.0%)	0(0.0%)
	CNS complications	3(3.1%)	2 (66.7%)	1 (33.3%)
	No complications	82 (80.2%)	81 (98.8%)	1(1.2%)
^*^Treatments	Oxygen	69 (71.88%)	67 (69.8%)	2 (100.0%)
	Steroid	13 (13.54%)	12 (12.5%)	1 (50.0%)
	Bronchodilator	10 (10.42%)	10 (10.4%)	0(0.0%)
	Hydroxychloroquine	10 (10.42%)	10 (10.4%)	0(0.0%)
	Antiviral	6 (6.25%)	5(5.2%)	1 (50.0%)
	Antibacterial	47 (48.96%)	45 (46.9%)	2 (100.0%)
**Discharge**	Home	94 (97.92%)	94 (100%)	0(0.0%)
	Dead	2 (2.08%)	0(0.0%)	2 (100.0%)

* *Percentage does not add up to 100 because more than one element was used*.

**Figure 1:**
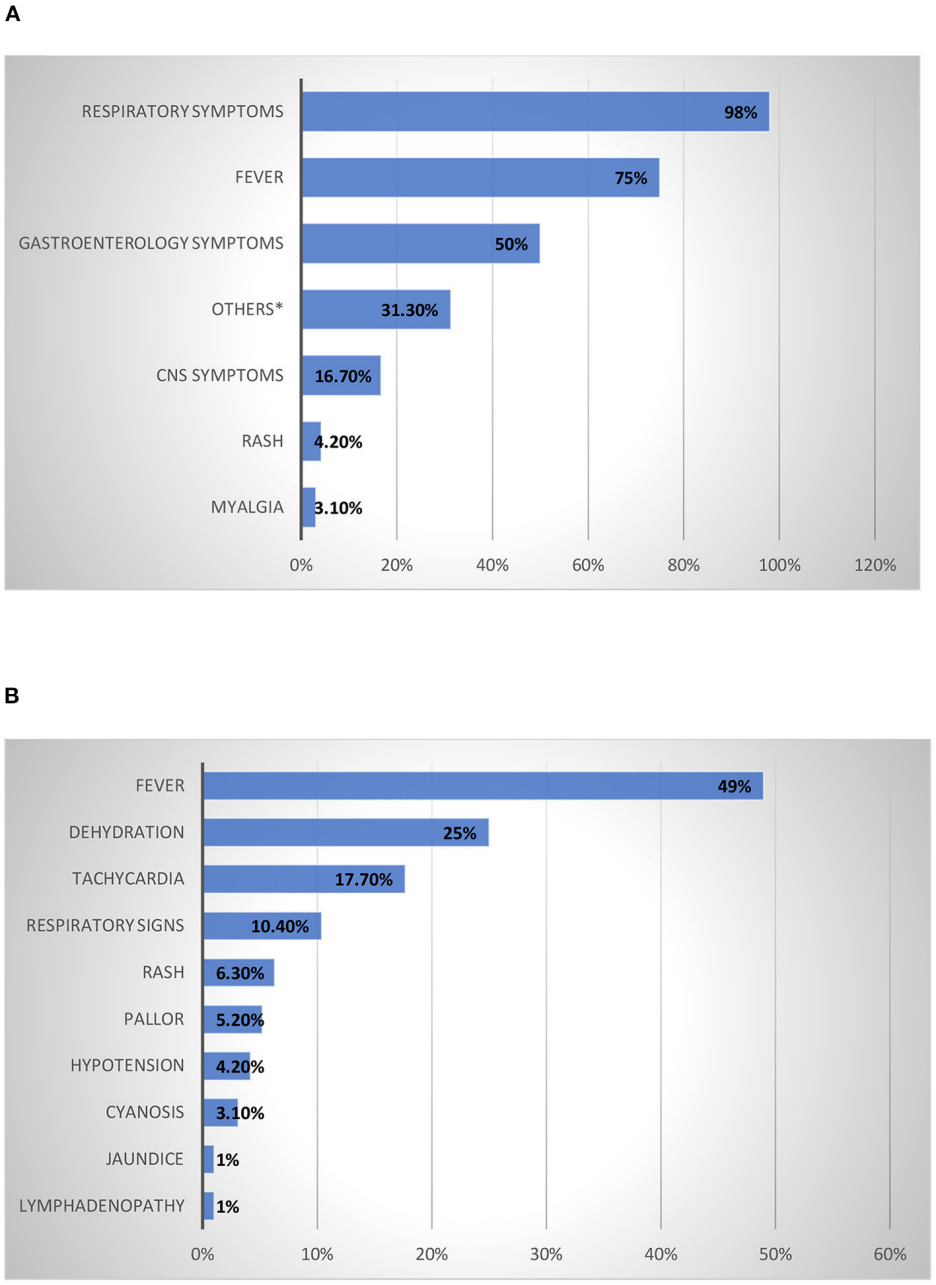
Presenting Symptoms and Physical Signs of Children Admitted with A Confirmed Diagnosis Of COVID-19. **(A)** Presenting symptoms of children admitted with a confirmed diagnosis of COVID-19 infection. *Decrease activity and decrease appetite. **(B)** Physical signs of children admitted with confirmed diagnosis of COVID-19 infection.

The presenting symptoms and physical signs of children admitted with a confirmed diagnosis of COVID-19 were investigated. The most common presenting symptoms were respiratory symptoms (98%), followed by fever (75%) and gastrointestinal symptoms (50%); the remaining symptoms were present in <50% of patients (Figure 1A). CNS symptoms as abnormal movement and decrease level of consciousness were found in 16% of the patients. Fever was the most reported physical sign (49%), followed by dehydration (25%), tachycardia (17.7%), and respiratory signs (10.4%), while the other signs were present in <10% of the patients (Figure 1B).

Of the 96 children admitted, 10 (10.42%) were critical and required PICU care, as shown in Table 2. Most of them required hospitalization for >10 days (60%), but the mean PICU length of stay was ≤4 days (70%). The minority of patients admitted to the PICU required intubation (20%). Most patients had COVID-19 from an unknown source (80%) and had comorbidities (70%). Abnormal physical signs such as dehydration (70%), tachycardia (50%), and respiratory signs (40%) were more common in children who required admission to the PICU than in those who did not. Pneumonia (40%) followed by central nervous system complications (30%) were the most common complications. All patients were treated with oxygen (100%), followed by antibacterial antibiotics (80%) and steroids (40%). Most patients fully recovered (90%) and were discharged home (90%). We report one patient died in the PICU.

**Table 2:** Patients demographics and disease characteristics with PICU vs. regular ward admission of children admitted with confirmed COVID-19 infection (*n* = 96).

**Variables**		**Total population *n* = 96 (%)**	**Admission**	
			**PICU%**	**Ward%**
			***n* = 10 (10.42%)**	***n* = 86 (89.58%)**
**Age**	≤3	60 (63.8%)	5 (50.0%)	55 (64.0%)
	>3	36 (36.20%)	5 (50.0%)	31 (36.0%)
**Sex**	Male	50 (52.1%)	8 (80.0%)	42 (48.8%)
	Female	46 (47.9%)	2 (20.0%)	(44 51.2%)
**Nationality**	Saudi	93 (96.9%)	10 (100.0%)	83 (96.5%)
	Non-Saudi	3(3.1%)	0(0.0%)	3(3.5%)
**Length of hospital stay**	≤10	70 (74.5%)	4 (40.0%)	66 (76.7%)
	>10	26 (25.5%)	6 (60.0%)	20 (23.3%)
**Pediatric intensive care unit length of stay**	≤4	7 (70.0%)	7 (70.0%)	
	>4	3 (30.0%)	3 (30.0%)	
**Intubation**	Yes	2 (2.08%)	2 (20.0%)	
	No	94 (97.92%)	8 (80.0%)	
**Duration of symptoms**	≤3	68 (70.83%)	5 (50.0%)	63 (73.3%)
	> 3	28 (29.17%)	5 (50.0%)	23 (26.7%)
**Possible source of infection**	Contact COVID-19 Case	40 (41.67%)	2 (20.0%)	38 (44.2%)
	Travel to Endemic Area	2 (2.08%)	0(0.0%)	2(2.3%)
	Unknown	54 (56.25%)	8 (80.0%)	46 (53.5%)
**Comorbidities**	Yes	47 (49.0%)	7 (70.0%)	40 (46.5%)
	No	49 (51.0%)	3 (30.0%)	46 (53.5%)
^*^Signs	Cyanosis	3 (3.1%)	0 (0.0%)	3 (3.5%)
	Fever	47 (49.0%)	8 (80.0%)	39 (45.3%)
	Dehydration	24 (25.0%)	7 (70.0%)	17 (19.8%)
	Hypotension	4(4.2%)	1 (10.0%)	3(3.5%)
	Pallor	5(5.2%)	1 (10.0%)	4(4.7%)
	Tachycardia	17 (17.7%)	5 (50.0%)	12 (14.0%)
	Respiratory Signs	10 (10.40%)	4 (40.0%)	6(7.0%)
	Lymphadenopathy	1(1.0%)	0(0.0%)	1(1.2%)
	Jaundice	1(1.0%)	0(0.0%)	1(1.2%)
	Rashes	6(6.3%)	0(0.0%)	6(7.0%)
^*^Complications	Secondary Pneumonia	10 (10.4%)	4 (40.0%)	6 (6.98%)
	ARDS	2(2.1%)	2 (20.0%)	0(0.0%)
	Multiorgan Failure	4(4.2%)	1 (10.0%)	3(3.5%)
	CNS Complications	3(3.1%)	3 (30.0%)	0(0.0%)
	No Complications	82 (80.2%)	4 (40.0%)	78 (90.7%)
^*^Treatments	Oxygen	69 (71.88%)	10 (100.0%)	59 (68.6%)
	Steroids	13 (13.54%)	4 (40.0%)	9 (10.5%)
	Bronchodilator	10 (10.42%)	3 (30.0%)	7(8.1%)
	Hydroxychloroquine	10 (10.42%)	3 (30.0%)	7(8.1%)
	Antiviral	6 (6.25%)	0(0.0%)	6(7.0%)
	Antibacterial	47 (48.96%)	8 (80.0%)	39 (45.3%)
**Outcome**	Full recovery	94 (97.92%)	9(90%)	85 (98.83%)
	Death	2 (2.08%)	1(10%)	1 (1.17%)
**Discharge**	Home	94 (97.92%)	9(90%)	85 (98.84%)
	Dead	2 (2.08%)	1(10%)	1 (1.16%)

* *Percentage does not add up to 100 because more than one element was used*.

The laboratory results and imaging findings were assessed, and their correlations with PICU and ward admissions are shown in Table 3. Among the patients admitted to the regular ward, 86 (89.58%) tended to have the following findings: normal ECG findings, 83 (96.5%); normal chest X-ray findings, 72 (83.7%); abnormal chest computed tomography findings, 2 (2.3%); normal white blood cell count, 55 (64%); normal neutrophil count, 61 (70.9%); normal hemoglobin level, 59 (68.6%); normal lymphocyte count, 46 (53.49%); normal alanine aminotransferase (ALT) level, 81 (94.19%); normal aspartate aminotransferase (AST) level, 81 (94.19%); normal creatinine level, 49 (56.98%); normal ferritin level, 65 (75.58%); high D-dimer level, 78 (90.7%); high CRP level, 62 (72.09%); and adenovirus-positive result, 1 (1.16%). Those with positive bacteria in the blood (3 [30%]), urine (3 [30%]), or tracheal aspirate [1 (10%)] were admitted to the PICU.

**Table 3:** Labs and images associated with pediatric intensive care unit vs. regular ward admission of children admitted with confirmed COVID-19 infection (*n* = 96).

**Variables**			**Total population *n* = 96 (%)**	**Admission**	
				**Pediatric Intensive Care Unit %**	**Ward %**
				***n* = 10 (10.42%)**	***n* = 86 (89.58%)**
**Imaging**	Electrocardiogram	Normal	92 (95.8%)	9 (90.0%)	83 (96.5%)
		Abnormal	2(2.1%)	0(0.0%)	2(2.3%)
		Not Done	2(2.1%)	1 (10.0%)	1(1.2%)
	Chest X-ray	Normal	76 (79.2%)	4 (40.0%)	72 (83.7%)
		Abnormal	20 (20.8%)	6 (60.0%)	14 (16.3%)
	Chest computed tomography scan	Normal	3(3.1%)	0(0.0%)	3(3.5%)
		Abnormal	3(3.1%)	1 (10.0%)	2(2.3%)
		Not Done	90 (93.8%)	9 (90.0%)	81 (94.2%)
**Laboratory**	White blood cells	Normal	61 (63.54%)	6 (60.0%)	55 (64.0%)
		High	27 (28.13%)	4 (40.0%)	23 (26.7%)
		Low	8 (8.33%)	0(0.0%)	8(9.3%)
	Neutrophils	Normal	67 (69.8%)	6 (60.0%)	61 (70.9%)
		High	22 (22.9%)	4 (40.0%)	18 (20.9%)
		Low	7(7.3%)	0(0.0%)	7(8.1%)
	Hemoglobin	Normal	65 (67.7%)	6 (60.0%)	59 (68.6%)
		High	1 (1.0%)	0(0.0%)	1(1.2%)
		Low	30 (31.3%)	4 (40.0%)	26 (30.2%)
	Lymphocytes	Normal	51 (53.1%)	5 (50.0%)	46 (53.49%)
		High	27 (28.1%)	4 (40.0%)	23 (26.74%)
		Low	18 (18.8%)	1 (10.0%)	17 (19.77%)
	Alanine aminotransferase	Normal	90 (93.8%)	9 (90.0%)	81 (94.19%)
		High	6(6.2%)	1 (10.0%)	5 (5.81%)
	Aspartate aminotransferase	Normal	89 (92.71%)	8 (80.0%)	81 (94.19%)
		High	6 (6.25%)	2 (20.0%)	4 (4.65%)
		Low	1 (1.04%)	0(0.0%)	1 (1.16%)
	Creatinine	Normal	54 (56.25%)	5 (50.0%)	49 (56.98%)
		High	13 (13.54%)	2 (20.0%)	11 (12.79%)
		Low	29 (30.21%)	3 (30.0%)	26 (30.23%)
	Ferritin	Normal	73 (76.04%)	8 (80.0%)	65 (75.58%)
		High	18 (18.75%)	2 (20.0%)	16 (18.60%)
		Low	5 (5.21%)	0(0.0%)	5 (5.81%)
	D.Dimer	Normal	7 (7.29%)	0(0.0%)	7 (8.14%)
		High	88 (91.67%)	10 (100.0%)	78 (90.70%)
		Low	1 (1.04%)	0(0.0%)	1 (1.16%)
	C-Reactive protein	Normal	7 (7.29%)	1 (10.0%)	6 (6.98%)
		High	69 (71.88%)	7 (70.0%)	62 (72.09%)
		Low	20 (20.83%)	2 (20.0%)	18 (20.9%)
	^*^Positive bacteria	Blood	5 (5.21%)	3(30%)	2 (2.32%)
		Urine	4 (4.17%)	3(30%)	1 (1.16%)
		Tracheal Aspirate	1 (1.04%)	1(10%)	0(0.0%)
**Viral respiratory**				
	Respiratory syncytial virus	Negative	23 (23.96%)	3(30%)	20 (23.26%)
		Not done	73 (76.04%)	7(70%)	66 (76.74%)
	Adeno virus	Negative	1 (1.04%)	3(30%)	19 (22.1%)
		Positive	22 (22.92%)	0(0.0%)	1 (1.16%)
		Not done	73 (76.04%)	7(70%)	66 (76.74%)
	Para influenza	Negative	23 (23.96%)	3(30%)	20 (23.26%)
		Not done	73 (76.04%)	7(70%)	66 (76.74%)
	Influenza	Negative	23 (23.96%)	3 (30%)	20 (23.26%)
		Not done	73 (76.04%)	7 (70%)	66 (76.74%)

* *Percentage does not add up to 100 because more than one element was used*.

Among the 96 children admitted with confirmed COVID-19 infection, four were found to have MIS-C. The majority of the children who had MIS-C were aged >3 years (75%), all patients were Saudi, stayed for ≤10 days in the hospital, experienced symptoms for >3 days, and had no comorbidities. Fever (100%), diarrhea and vomiting (75%), and abdominal pain with decreased activity and appetite were the most prevalent presenting symptoms (50%). The most common abnormal physical signs in individuals with MIS-C were fever (100%), rash (50%), tachycardia (50%), hypotension (50%), and dehydration (50%). All patients with MISC developed multiple organ failure and received antibacterial treatment, and most of them received oxygen (50%). Generally, these patients did not require PICU admission (75%); all of them fully recovered in <10 days (100%) and were discharged home (100%).

Out of the 96 admitted children with confirmed SARS CoV-2 infection, we reported two deaths (2.08%), one in the ward due to respiratory cause and the other in the PICU due to complications of the disease. The two cases were aged >3 years old (100%), male (100%), Saudi (100%), stayed for >10 days in the hospital (100%) (Tables 1, 2). One of the cases infected due to contact with COVID-19 infection and the other infected with unknown source. In addition, both cases known to have comorbidities as heart disease, renal disease, and neuromuscular disease. Fever and sore throat associated with cough, shortness of breath, and altered level of consciousness were commonly presented in both cases. Moreover, one of the cases developed complications of COVID-19 infection as secondary pneumonia, acute respiratory distress syndrome, and central nervous system complications whereas, the other case has no complications. Anemia, high creatinine, and high D.dimer were found in the both cases yet, one of them has positive blood culture. Although, both cases treated with oxygen, and antibacterial. However, one of the cases also treated with antiviral and steroids.

## Discussion

This is one of the retrospective studies in SA on the clinical features of pediatric patients with COVID-19. Studies published in late 2020 and 2021 were conducted in the SA and Gulf regions (5, 15, 22–24). However, three previous retrospective studies were published early in 2021: one retrospective study included 62 children from a single center in Jeddah, SA (25), the other conducted in a tertiary hospital in Riyadh included 742 pediatric inpatients and outpatients (6), and the last one conducted in three tertiary academic hospitals in SA enrolled 88 hospitalized children, the majority of whom had mild to moderate diseases (7). All the studies demonstrated that most patients had mild to moderate disease, and this may be due to the strict protocol endorsed by the SMOH (6, 7, 25). On the other hand, our study showed that the majority of hospitalized pediatric patients had severe COVID-19 infection according to the SMOH definition by requiring oxygen (71.88%). Fever and respiratory symptoms were also shown to be the most common clinical characteristics of COVID19 in children. Infants were more likely than other age groups to experience dyspnea. There have also been reports of gastrointestinal symptoms such as diarrhea and vomiting. Multiple studies have produced similar results (6, 7, 25).

Children with COVID-19 have been reported to more often have mild clinical symptoms than adults (26–28). In a review of studies regarding COVID-19 in children in SA and worldwide, most children with COVID-19 had mild symptoms, a good prognosis, and recovered within 1–2 weeks (6, 7, 25, 29). Our retrospective review showed that most of the patients had severe symptoms since oxygen was required in almost 71.88% of the cases, and they recovered within ≤3 days with good prognosis. Xiaoxia et al. (30) provided a summary of 171 confirmed pediatric cases in China, showing that most infected children had a mild clinical course, and asymptomatic infections were common (15.8%). There was one death involving a 10-month-old child with intussusception and multiorgan failure (30). In a large retrospective cohort study of children with COVID-19 in China, 4 and 51% of the patients were diagnosed without any symptoms or with mild symptoms, respectively, and only one child was reported to have died (1). A small percentage of patients in our study experienced complications, such as secondary pneumonia (10.4%) or acute respiratory distress syndrome (2.1%). Furthermore, the prevalence of severe and critical cases among infants (63.8%) was higher than in other age groups. Another retrospective study reported two critical pediatric patients aged approximately 1 year who required invasive mechanical ventilation, corticosteroids, and immunoglobulin therapy (31). However, our study found that only a minority of patients (10.42%) required PICU care, and two patients died. This shows that pediatric patients experience less severe illnesses than adults; nevertheless, young children, particularly infants, might have higher risk of sever COVID-19 infection than older children (1). One report from the United States (32) demonstrated that 73% of pediatric patients had symptoms of fever, cough, or shortness of breath as compared with 93% of adults. Between 5.7 and 20% of pediatric patients were reported to be hospitalized, with 0.58–2.0% admitted to the ICU. Two deaths were reported in this cohort of pediatric patients. Similar results have been reported in multiple studies (6, 7, 25). These findings show that children experience respiratory symptoms less frequently than adults. Although the majority of pediatric patients with COVID-19 did not have a severe illness, a serious COVID-19 could lead to ICU admission and even death in children.

The main radiological features in pediatric patients with COVID-19 have been reported to be subpleural ground-glass opacities and consolidations with surrounding halo signs, suggestive of pneumonia (28, 33). The majority of children with these findings were asymptomatic or had only minor symptoms. Additionally, Qiu et al. (26) reported that the prevalence of pneumonia with COVID-19 (53%) was higher than that with H1N1 influenza (11%). However, our study demonstrated that the majority of patients had normal radiological findings, while a minority demonstrated pneumonic shadow with no typical findings or correlations with severity. Similar results have been reported in a couple of studies (7, 25).

Typical abnormal laboratory findings did not show significant differences among different cohorts. Xia et al. (31) reported laboratory findings in pediatric patients with COVID-19, including lymphopenia (35%) and elevated ALT (25%), creatine kinase-MA (75%), CRP (45%), and procalcitonin (80%) levels. The main laboratory findings found in our study were lymphopenia (18.8%) and elevated CRP (71.88%), D-dimer (91.67%), ALT (6.2%), and AST (6.25%) levels. A study from SA reported lymphopenia and high D-dimer and CRP levels in most cases (25). Another retrospective study conducted in SA showed that patients who were admitted to the PICU had higher laboratory ALT, AST, and creatinine levels at admission than those who were not admitted to the PICU (6). Further, a study of adult patients with COVID-19 demonstrated that prothrombin time and D-dimer levels were higher in patients admitted to the ICU than those who were not (34). Zhou et al. (35) reported that an elevated D-dimer level was one of the important risk factors of death in adult patients after COVID-19. Our study showed high D-dimer levels in all PICU-admitted cases (100%).

Despite the fact that most children with COVID-19 have a mild course, some develop an extensive systemic inflammatory response known as MISC, which is similar to KD and can affect numerous organs, including the gastrointestinal system, skin, lymph nodes, and others; it can manifest with shock, cardiac dysfunction, acute heart failure, and extremely high levels of inflammatory biomarkers and the brain natriuretic peptide (36–38). Almoosa et al. (39) reported 10 cases of MIS-C in the eastern area of SA with wide-spectrum diseases and eventually with good outcomes. Only four patients in our cohort with MISC-associated COVID-19 developed a severe inflammatory phenotype, including fever, gastrointestinal symptoms, shock, cardiac dysfunction, liver and kidney dysfunction, and coagulopathy, as well as clinical features that overlapped with KD, such as rash, conjunctivitis, swelling of the extremities, and oropharyngeal redness; all patients recovered without sequelae and responded well to usual treatments, such as immunoglobulin or steroid therapy.

Protocols have changed periodically as evidence has arisen regarding the treatment of COVID-19. Many trials were conducted during the beginning of the pandemic, and at the time of writing this paper, no Food Drug Administration-approved drugs for the treatment of COVID-19 have been released. In our study, most of the cases were severe and pediatric patients recovered with oxygen (71.88%) as an interventional treatment followed by antibacterial antibiotics (48.96%).

The implication for practice of this study will enlighten and increase the knowledge of the health care providers about the disease unique characteristics and their outcomes to detect it early in order to deliver the optimum management specially for the critically ill patients. Also, awareness campaigns for the public on keeping all preventive measures, such as wearing face masks, social distancing, and complying with good hand hygiene practice, will prevent the spread of the infection. Additionally, the importance of vaccination in pediatric age groups is crucial.

## Conclusions

In conclusion, our findings showed that the majority of the admitted children were ≤3 years of age (52.1%) and infected with an unknown source (56.2%). Moreover, the majority of the cases had severe COVID-19 infection as they required oxygen (71.88%). In addition, the patients presenting with respiratory symptoms and fever were associated with high CRP and D-dimer levels, but most of them had a favorable outcome (97.92%). However, some cases were critical and resulted in death. Early detection of COVID-19's unique features in pediatric patients and quick treatment are vital for critically ill patients. Due to the continuous spread of the virus and increasing infection rate, future studies will be critical to better understanding the disease spectrum and potential therapeutic options.

### Limitations

The results represent an early stage of COVID-19 infection and transmission at one site, and we only had a small number of critical cases, which is one of the study's major limitations. In addition, this study was done during the first wave of the pandemic and that other variants of COVID-19 have appeared since then. Moreover, a number of significant elements changed over this time, including the implementation of social distancing, national lock-down measures, and increased testing speed, all of which could have altered the true incidence and outcome data. In addition, revisions in the SMOH's recommendations might have affected the results. Lastly, as a retrospective cohort study, it was difficult to maintain a long follow-up period, so the study is susceptible to some loss of data—we excluded five patients with incomplete data. Moreover, the lack of a control group (not exposed) to compare with the exposed group so the relative risk of the exposure can be calculated.

## Data Availability Statement

The raw data supporting the conclusions of this article will be made available by the authors, without undue reservation.

## Ethics Statement

The studies involving human participants were reviewed and approved by King Fahad Medical City. Written informed consent to participate in this study was provided by the participants' legal guardian/next of kin.

## Author Contributions

AA: conceptualization, investigation, writing—original draft, writing—review and editing, visualization, and project administration. DA: conceptualization, investigation, writing—review and editing, visualization, and project administration. SAlG, SAlF, and EA: investigation, methodology, and writing—review and editing. YB: formal analysis, data curation, and writing—review and editing. TA and LA-A: methodology, investigation, and writing—review and editing. MA: methodology and investigation. YA: formal analysis, resources, writing—original draft, writing—review and editing, supervision, and project administration. All authors contributed to the article and approved the submitted version.

## Conflict of Interest

The authors declare that the research was conducted in the absence of any commercial or financial relationships that could be construed as a potential conflict of interest.

## Publisher's Note

All claims expressed in this article are solely those of the authors and do not necessarily represent those of their affiliated organizations, or those of the publisher, the editors and the reviewers. Any product that may be evaluated in this article, or claim that may be made by its manufacturer, is not guaranteed or endorsed by the publisher.

## References

[1] Dong Y Mo X Hu Y Qi X Jiang F Jiang Z. Epidemiology of COVID-19 among children in China. Pediatrics. (2020) 145:e20200702. 10.1542/peds.2020-0702 32179660

[2] Hoang A Chorath K Moreira A Evans M Burmeister-Morton F Burmeister F. COVID-19 in 7780 pediatric patients: a systematic review. EClinicalMedicine. (2020) 24:100433 10.1016/j.eclinm.2020.100433 32766542 PMC7318942

[3] Lingawi HS. Covid-19 in children: prevalence, clinical characteristics, severity, and transmission. J Res Med Dent Sci. (2021) 9:24–30.33938934

[4] Alsohime F Temsah MH Al-Nemri AM Somily AM Al-Subaie S. COVID-19 infection prevalence in pediatric population: etiology, clinical presentation, and outcome. J Infect Public Health. (2020) 13:1791–6. 10.1016/j.jiph.2020.10.008 33127335 PMC7574780

[5] Hasan MR Al Zubaidi K Diab K Hejazi Y Bout-Tabaku S Al-Adba B. COVID-19 related multisystem inflammatory syndrome in children (MIS-C): a case series from a tertiary care pediatric hospital in Qatar. BMC Pediatr. (2021) 21:267. 10.1186/s12887-021-02743-8 34103044 PMC8185322

[6] Alharbi M Kazzaz YM Hameed T Alqanatish J Alkhalaf H Alsadoon A. SARS-CoV-2 infection in children, clinical characteristics, diagnostic findings and therapeutic interventions at a tertiary care center in Riyadh, Saudi Arabia. J Infect Public Health. (2021) 14:446–53. 10.1016/j.jiph.2020.12.034 33765595 PMC7833958

[7] Kari JA Shalaby MA Albanna AS Alahmadi TS Sukkar SA MohamedNur HAH. Coronavirus disease in children: a multicentre study from the Kingdom of Saudi Arabia. J Infect Public Health. (2021) 14:543–9. 10.1016/j.jiph.2021.01.011 33756192 PMC7981189

[8] Sheshah E Sabico S Albakr R Sultan A Alghamdi K Al Madani K. Prevalence of diabetes, management and outcomes among covid-19 adult patients admitted in a specialized Tertiary Hospital in Riyadh, Saudi Arabia. Diabetes Res Clin Pract. (2021) 172:108538. 10.1016/j.diabres.2020.108538 33189790 PMC7661919

[9] Alguwaihes AM Al-Sofiani ME Megdad M Albader SS Alsari MH Alelayan A. Diabetes and covid-19 among hospitalized patients in Saudi Arabia: a single-centre retrospective study. Cardiovasc Diabetol. (2020) 19:205. 10.1186/s12933-020-01184-4 33278893 PMC7718833

[10] Alguwaihes AM Sabico S Hasanato R Al-Sofiani ME Megdad M Albader SS et al. Severe vitamin D deficiency is not related to SARS-COV-2 infection but may increase mortality risk in hospitalized adults: a retrospective case–control study in an arab gulf country. Aging Clin Exp Res. (2021) 33:1415–22. 10.1007/s40520-021-01831-0 33788172 PMC8009930

[11] Alsofayan YM Althunayyan SM Khan AA Hakawi AM Assiri AM. Clinical characteristics of COVID-19 in Saudi Arabia: a national retrospective study. J Infect Public Health. (2020) 13:920–5. 10.1016/j.jiph.2020.05.026 32534945 PMC7833063

[12] Weqaya Public Health Authority. Saudi Center for Disease Prevention (2021).

[13] Saudi Ministry of Health. Coronavirus Disease 19 Guidelines (2020).

[14] Centers for Disease Control and Prevention. Multisystem inflammatory syndrome in children (MIS-C) associated with coronavirus Disease 2019 (COVID-19) (2020).

[15] Kobeisy SAN Harbi SA Mehdawi RS Bashammakh DS. Pediatric COVID-19 patients in Jeddah, Saudi Arabia: clinical, laboratory and radiological aspects. J Biomed Sci. (2020) 9. 10.36648/2254-609X.9.3.7

[16] Centers for Disease Control and Prevention. Trends in disease severity and health care utilization during the early Omicron variant period compared with previous SARS-COV-2 high transmission periods - United States, December 2020–January 2022. MMWR Morb Mortal Wkly Rep. (2022) 71:146–52. 35085225 10.15585/mmwr.mm7104e4 PMC9351529

[17] Temsah M-H Aljamaan F Alenezi S Alhasan K Alrabiaah A Assiri R. SARS-COV-2 omicron variant: exploring healthcare workers' awareness and perception of vaccine effectiveness: a national survey during the first week of WHO variant alert. Front Pub Health. 10:878159. 10.3389/fpubh.2022.878159 35400032 PMC8989964

[18] Temsah M Alenezi S Alarabi M Aljamaan F Alhasan K Assiri R. Healthcare Workers' SARS-COV-2 omicron variant uncertainty-related stress, resilience, and coping strategies during the first week of the world health organization's alert. Int J Environ Res Public Health. (2022) 19:1944. 10.3390/ijerph19041944 35206135 PMC8872197

[19] Al-Tawfiq J Hoang V-T Bui N Chu D-T Memish Z. The emergence of the Omicron (b.1.1.529) SARS-COV-2 variant: what is the impact on the continued pandemic? J Epidemiol Glob Health. (2022) 1:1–4. 10.1007/s44197-022-00032-w 35089588 PMC8795715

[20] Pediatric multisystem inflammatory Syndrome temporally associated With COVID-19 (PIMS) - Guidance for clinicians (2020) RCPCH.

[21] World Health Organization. Multisystem Inflammatory Syndrome in Children and Adolescents With Covid-19.

[22] Shaiba LA Altirkawi K Hadid A Alsubaie S Alharbi O Alkhalaf H. Covid-19 disease in infants less than 90 days: case series. Front Pediatr. (2021) 9:674899. 10.3389/fped.2021.674899 34322461 PMC8311174

[23] Ennab F ElSaban M Khalaf E Tabatabaei H Amar HK Devi BR. Clinical characteristics of children with covid-19: a multicenter study in the United Arab Emirates. J Pediatr Infect Dis Soc. (2021) 10:S17–8. 10.1093/jpids/piab031.039 34643535 PMC8575012

[24] Al Mansoori L Al Kaabi S Nair SC Al Katheeri M Ghatasheh G Al Dhanhani H. Epidemiological characteristics of children with coronavirus at a joint commission-accredited hospital in the United Arab Emirates. J Fam Med Prim Care. (2021) 10:2348–52. 10.4103/jfmpc.jfmpc_2161_20 34322436 PMC8284226

[25] Alnajjar AA Dohain AM Abdelmohsen GA Alahmadi TS Zaher ZF Abdelgalil AA. Clinical characteristics and outcomes of children with COVID-19 in Saudi Arabia. Saudi Med J. (2021) 42:391–8. 10.15537/smj.2021.42.4.20210011 33795494 PMC8128626

[26] Qiu H Wu J Hong L Luo Y Song Q Chen D. Clinical and epidemiological features of 36 children with coronavirus disease 2019 (COVID-19) in Zhejiang, China: an observational cohort study. Lancet Infect Dis. (2020) 20:689–96. 10.1016/S1473-3099(20)30198-5 32220650 PMC7158906

[27] Tagarro A Epalza C Santos M Sanz-Santaeufemia F Otheo E Moraleda C. Screening and severity of COVID-19 in children in Madrid, Spain. JAMA Pediatr. (2021) 175:316–7. 10.1001/jamapediatrics.2020.1346 32267485 PMC7142799

[28] Chen ZM Fu JF Shu Q Chen YH Hua CZ Li FB. Diagnosis and treatment recommendations for pediatric respiratory infection caused by the 2019 novel coronavirus. World J Pediatr. (2020) 16:240–6. 10.1007/s12519-020-00345-5 32026148 PMC7091166

[29] Castagnoli R Votto M Licari A Brambilla I Bruno R Perlini S. Severe acute respiratory syndrome coronavirus 2 (SARS-CoV-2) infection in children and adolescents: a systematic review. JAMA Pediatr. (2020) 174:882–9. 10.1001/jamapediatrics.2020.1467 32320004

[30] Xiaoxia L Liqiong Z Hui D Jingjing Z Yuan YL Jingyu Q. SARS-CoV-2 infection in children. N Engl J Med. (2020) 382:1663–5. 10.1056/NEJMc2005073 32187458 PMC7121177

[31] Zheng F Liao C Fan QH Chen HB Zhao XG Xie ZG. Clinical characteristics of children With coronavirus disease 2019 In Hubei, China. Current Med Sci. (2020) 40:275–80. 10.1007/s11596-020-2172-6 32207032 PMC7095065

[32] Bialek S Gierke R Hughes M McNamara L Pilishvili T Skoff T. Coronavirus disease 2019 in children - United States, February 12 – april 2, 2020. MMWR Morb Mortal Wkly Rep. (2020) 69:422–6. 10.15585/mmwr.mm6914e4 32271728 PMC7147903

[33] Xia W Shao J Guo Y Peng X Li Z Hu D. Clinical and CT features in pediatric patients With covid-19 infection: different points from adults. Pediatr Pulmonol. (2020) 55:1169–74. 10.1002/ppul.24718 32134205 PMC7168071

[34] Huang C Wang Y Li X Ren L Zhao J Hu Y. Clinical features of patients infected with 2019 novel coronavirus in Wuhan, China. Lancet. (2020) 395:497–506. 10.1016/S0140-6736(20)30183-5 31986264 PMC7159299

[35] Zhou F Yu T Du R Fan G Liu Y Liu Z. Clinical course and risk factors for mortality of adult inpatients with COVID-19 IN Wuhan, China: a retrospective cohort study. Lancet. (2020) 395:1054–62. 10.1016/S0140-6736(20)30566-3 32171076 PMC7270627

[36] Verdoni L Mazza A Gervasoni A Martelli L Ruggeri M Ciuffreda M. An outbreak of severe Kawasaki-like disease at the Italian epicentre of the SARS-CoV-2 epidemic: an observational cohort study. Lancet. (2020) 395:1771–8. 10.1016/S0140-6736(20)31103-X 32410760 PMC7220177

[37] Whittaker E Bamford A Kenny J Kaforou M Jones C Shah P. Clinical characteristics of children with sars-cov-2–associated pediatric inflammatory multisystem syndrome. JAMA. (2020) 324:259–69. 10.1001/jama.2020.10369 32511692 PMC7281356

[38] Cheung EW Zachariah P Gorelik M Boneparth A Kernie SG Orange JS. Multisystem inflammatory syndrome related to COVID-19 in previously healthy children and adolescents in New York City. JAMA. (2020) 324:294–6. 10.1001/jama.2020.10374 32511676 PMC7281352

[39] Almoosa ZA Al Ameer HH AlKadhem SM Busaleh F AlMuhanna FA Kattih O. Multisystem inflammatory syndrome in children, the real disease of COVID-19 in pediatrics - a Multicenter case series from AL-AHSA, Saudi Arabia. Cureus. (2020) 12:e11064. 10.7759/cureus.11064 33240687 PMC7682634

